# School-based group interpersonal therapy for adolescents with depression in Nepal: protocol for a phase III realist cluster-randomised controlled trial

**DOI:** 10.1186/s12888-025-07302-4

**Published:** 2025-09-25

**Authors:** Kelly Rose-Clarke, Cemile Ceren Sonmez, Sujan Shrestha, Bishnu Lamichhane, Indira Pradhan, Parbati Pandey, Pratima Kandel, John Hodsoll, Lauren Yan, Bryan Patenaude, Helen Verdeli, Kamal Gautam, Mark Jordans, Chris Bonell, Nagendra P. Luitel

**Affiliations:** 1 UCL Institute for Global Health, University College London, 30 Guildford Street, London, WC1N 1EH UK; 2 Transcultural Psychosocial Organization Nepal, Kathmandu, Nepal; 3 Institute of Psychiatry, Psychology and Neuroscience, Kings College London, London, UK; 4 Johns Hopkins Bloomberg School of Public Health, Baltimore, US; 5 Teachers College, Columbia University, New York, US; 6 Faculty of Public Health and Policy, London School of Hygiene and Tropical Medicine, London, UK

**Keywords:** Adolescents, Depression, Interpersonal therapy, Nepal, Realist randomised controlled trial, Cluster randomised controlled trial

## Abstract

**Background:**

Depression is a leading cause of disability among adolescents, with the burden disproportionately affecting low- and middle-income countries (LMICs) where access to mental health care is limited. Interpersonal therapy (IPT), a structured psychological intervention, has shown promise in treating adolescent depression but there is limited evidence from LMICs and research on how it works and in which contexts it works best. This protocol describes a realist cluster-randomised controlled trial (cRCT) assessing the effectiveness, cost-utility and mechanisms of school-based group IPT for adolescents with depression in Nepal.

**Methods:**

This superiority phase III cRCT will be conducted in 48 public secondary schools across Chitwan and Nawalpur districts, with schools randomised 1:1 to intervention or enhanced usual care. Adolescents aged 13–19 with depression (Patient Health Questionnaire modified for adolescents, PHQ-A score ≥11) will be recruited from grades 7–9. The intervention comprises two individual and ten weekly group IPT sessions delivered by trained lay facilitators. Adolescents will be surveyed pre-randomisation (baseline) and five (midline), 17 (endline) and 32 weeks (follow-up) post randomisation. The primary outcome is depression severity at 17 weeks post-randomisation assessed using the PHQ-A. Secondary outcomes include anxiety, post-traumatic stress disorder, functional impairment, school attendance and quality of life. Intermediate outcomes including hope, emotion regulation, and social support will be assessed to examine mechanisms of change. A priori hypotheses concerning IPT’s mechanisms and contextual factors influencing these (context-mechanism-outcome configurations) will be refined through analysis of qualitative process data and tested in mediation, moderation and moderated mediation analyses of trial data. Economic evaluation will estimate cost-utility and benefit-cost ratios from both provider and modified societal perspectives. The process evaluation will assess fidelity, reach, and acceptability in various school settings.

**Discussion:**

This trial is the first to integrate realist evaluation into a cRCT of a psychological intervention for adolescents in a LMIC and has potential to advance research and practice by elucidating how IPT works in a real-world context. If IPT is effective in Nepal, it could be scaled up through the education system as a part of a comprehensive school mental health care package.

**Trial registration:**

ISRCTN52852397 (registered 21/03/2025).

## Background

Globally, depression is the second highest cause of years lived with disability and the burden is increasing, especially among adolescents aged 10–19 [1–3]. When depression presents in adolescence it can have severe consequences for adolescents’ health and development including lower educational attainment, unemployment and increased risk of non-communicable disease in adulthood [4–6]. The burden of depression is highest in low- and middle-income countries (LMICs) where most of the world’s adolescents live and where there is the least access to mental health care [7].

Psychological interventions are first line treatments for adolescent depression. Among children and adolescents, psychological interventions have medium to large effects on depression but there is limited research from LMICs [8, 9]. Efforts to expand the evidence base in LMICs have focused on translating existing psychological interventions, often developed in a high-income country, but results have been mixed: an intervention shown to work in one setting does not always work elsewhere [10]. This is partly due to our limited understanding about how psychological interventions work. One prominent theory is that there are general mechanisms (“common factors” such as alliance, empathy, cultural adaptation) as well as mechanisms specific to certain intervention types [11]. However, research on these mechanisms has primarily been correlational, and there is a dearth of research on mechanisms conducted with populations in LMICs [12].

### Interpersonal therapy

Interpersonal therapy (IPT) is a manualised, time-limited psychological intervention for depression, implemented around the world. IPT focuses on four problems thought to trigger depression: grief, disputes, role transitions and social isolation. In IPT, individuals are encouraged to analyse and improve interpersonal relationships using techniques and strategies including linking mood to event and event to mood, role play and skill building. A meta-analysis of IPT for adolescents reported large reductions in depression (d = 1.48, *p* <.0001) [13]. IPT can be delivered by non-specialists in groups and is feasible in low-resource settings, but the evidence is inconsistent. In South Africa, group IPT for HIV positive adults reduced depressive symptoms compared to treatment as usual, but 21/41 participants allocated to IPT did not take it up [14]. In Uganda, group IPT for adolescents in internally displaced person camps improved depression among girls but not boys [15]. These findings highlight the need for research on IPT’s mechanisms and the contextual factors that influence them.

Between 2018 and 2022, we adapted the World Health Organization (WHO) group IPT manual for adolescents with depression in Nepal [16]. This involved translating the manual and reviewing it with Nepali mental health practitioners; conducting literature reviews on existing adaptations of IPT and concepts of mental health and interpersonal problems in Nepal; qualitative research with adolescents, caregivers, teachers and community health workers; consulting with a youth advisory board; and piloting the adapted manual with IPT trainers and facilitators [17, 18]. Key adaptations included integrating IPT into secondary schools, having separate groups for boys and girls, adding components to promote parental engagement and using locally acceptable terms for mental illness such as *man ko samasya* (heart-mind problem). We undertook an uncontrolled feasibility study of the intervention with 62 boys and girls aged 13–19 in Sindhupalchowk district, Nepal [19]. Adolescents attended 82.3% (standard deviation 18.9%) of group sessions. Depression improved between baseline and follow-up at 8–10 weeks post IPT: the Depression Self Rating Scale (DSRS) score decreased from 17.2 (95% confidence interval [CI]: 16.5–18,0) to 9.5 (95% CI 8.5–10.6) [19]. The estimated intervention unit cost was USD 96.9 with facilitators operating at capacity.

### Realist randomised controlled trial

In 2022, we started a 5-year programme to evaluate IPT in Nepal using a phase III realist randomised controlled trial (RCT) design. This design integrates realist evaluation into a traditional RCT design [20]. Realist evaluation seeks to understand the mechanisms through which social programmes work and why they work better in some contexts than others [21]. To do this, realist evaluators build programme theory from which they derive hypotheses about what intervention mechanisms operate, the contexts in which they operate and the outcomes that are generated. These so-called context-mechanism-outcome configurations (CMOCs) are then empirically tested through analyses of qualitative and quantitative data collected during programme implementation.

The advantage of realist RCTs over traditional RCTs is that they can provide the least biased estimate of intervention impact at population level and at the same time open the intervention “black box” to understand mechanisms of action and how they are influenced by context in terms of place and person characteristics [22, 23].

Combining realist and RCT approaches is not without criticism. Some realist evaluators argue the two approaches are epistemologically and ontologically incompatible, and that RCTs cannot generate the kind of data required to answer realist questions. However, these concerns are addressed in various publications, and an increasing number of real-world examples demonstrate the philosophical congruity, feasibility and usefulness of realist RCTs [23, 24].

Bonell and colleagues describe a methodological framework for conducting realist RCTs [22]. The first stage, prior to the trial, involves elaborating a theory of change and developing CMOCs. The second stage, whilst the trial is ongoing, uses qualitative process data to refine the CMOCs. In the third stage, outcome data are used to estimate the effect of the intervention at the population level, and quantitative process and outcome data are integrated to test the CMOCs via various statistical and other analyses. The end products are an evaluation of the intervention in context and an evidence-based theory of change.

### Formative research and pilot trial

We drew on Bonell’s framework in the design of our realist RCT, expanding the first stage to include formative research to generate a preliminary theory of change and initial CMOCs, and a pilot trial to refine CMOCs and test trial procedures. Formative research involved: a realist review of adolescent psychological interventions in LMICs; a review of moderators and mediators of IPT to synthesise evidence on intervention mechanisms and contextual factors; workshops with IPT practitioners across the globe to build a transcultural IPT theory of change for adolescents with depression; and a qualitative analysis of interview transcripts with participants and facilitators from the feasibility study in Nepal [25].

The pilot trial (ISRCTN14652885, protocol available online) was a parallel two-arm pilot cluster-RCT (cRCT) with schools as the unit of clustering. Baseline data were collected before randomisation. The control condition was enhanced usual care. We assessed participants in intervention and control arms at baseline, after the second group session (midline 1), after the sixth group session (midline 2), at endline (within two weeks of the final group session) and at follow-up (12 weeks after the final group session). We included adolescent boys and girls aged 13–19 from grades 8, 9 and 11, with depression defined as a score of 11 or more on the Patient Health Questionnaire modified for adolescents (PHQ-A) and 4 or more on a local measure of functional impairment. Mental health outcomes were depression, anxiety and post-traumatic stress disorder (PTSD). The sample comprised 161 adolescents (81 intervention, 80 control) from eight schools.

We set and met a priori progression criteria related to implementation, acceptability, fidelity, serious adverse events (SAEs), eligibility, missing data and participant retention. Depression scores at endline and follow-up were lower in the intervention arm compared to control at endline and follow-up, and we found significant improvements in functional impairment, anxiety and PTSD (manuscript in preparation). We observed reductions in interpersonal conflict in the intervention arm compared to control at midline 2, endline and follow-up, emotion regulation at midline 1 and follow-up and social support at midline 2. We did not find significant differences for self-efficacy, hope or interpersonal psychology skills.

### Theory of change and initial CMOCs

**Fig. 1:**
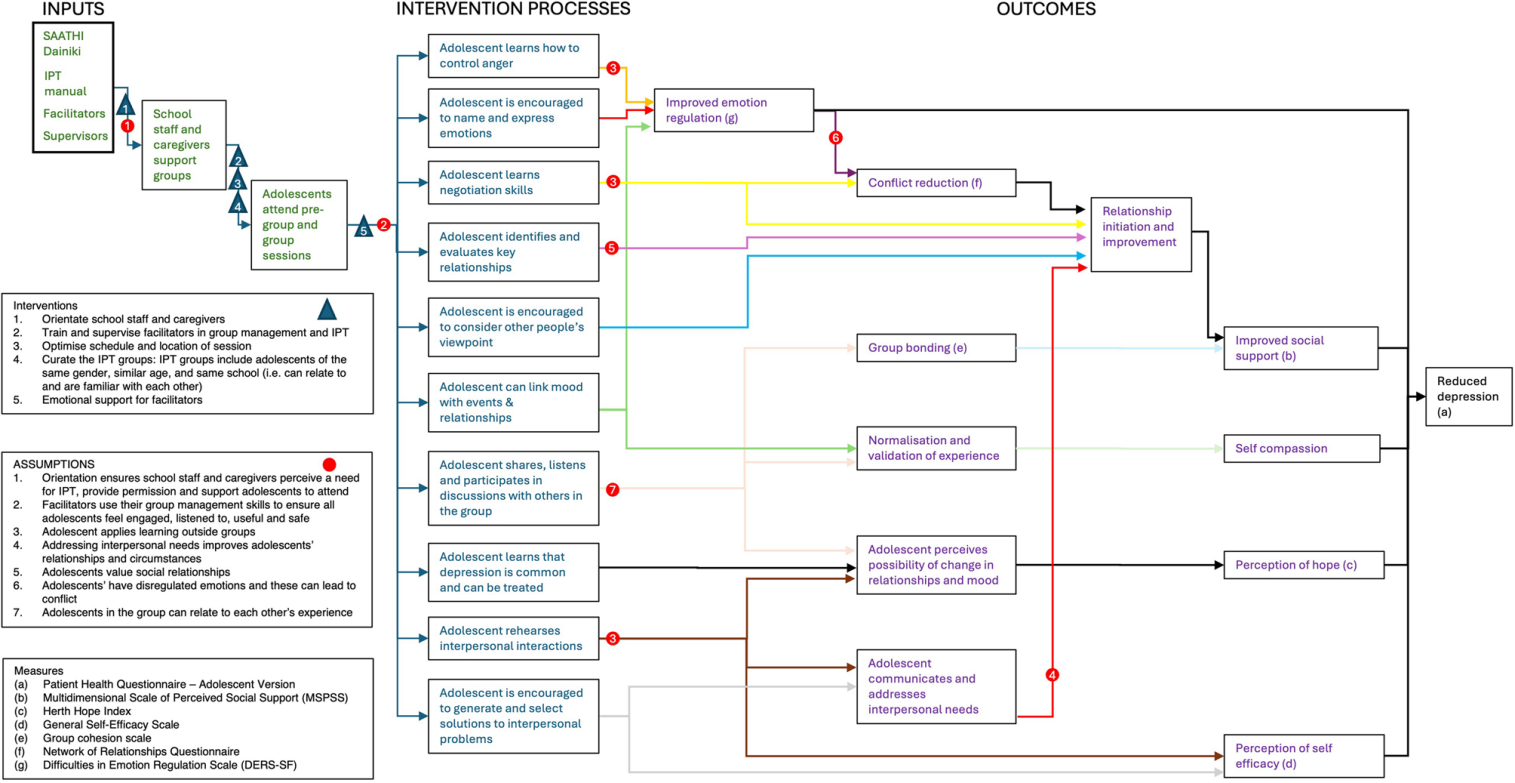
Theory of change for group IPT for adolescents with depression in Nepal

Through the formative research and pilot RCT, we developed a theory of change for IPT (Fig. 1), which includes roles for facilitators (delivering therapy and managing groups), school staff and caregivers (supporting adolescents) and adolescents (attending and participating in group sessions). Intervention mechanisms incorporate: social learning theory (adolescents acquire communication skills of sharing, listening, giving feedback, and negotiating); attachment theory (adolescents identify and evaluate key interpersonal relationships); mental health literacy (adolescents understand depression is common and can be treated); and problem-solving [26, 27]. Through these mechanisms, adolescents perceive the possibility of an improvement in their interpersonal circumstances and mood, internalise their membership of the IPT group (social identity theory), and improve their ability to empathise and communicate their interpersonal needs (emotion validation) [28]. These mechanisms generate intermediate impacts (relationship initiation, improved social support, conflict reduction, perception of hope, self-efficacy and emotion regulation), which in turn generate a reduction in depressive symptoms.

Based on the formative research and prior to the pilot cRCT, we formulated five CMOCs derived from our theory of change. We hypothesised that IPT would be more effective for adolescents from higher socioeconomic backgrounds who were not facing structural adversity because they would have more opportunities to apply the strategies they learned (CMOC1). Similarly, we posited that adolescents in schools with positive climates—characterised by strong relationships and mutual respect among teachers and students —would benefit from IPT because the school environment would support implementation of IPT strategies (CMOC2). We hypothesised that boys who were not constrained by harmful cultural norms or structural violence would be more able than girls to engage with and use the skills they learned in IPT (CMOC3), and that older and more cognitively able adolescents would be more capable of understanding and applying IPT strategies (CMOC4). We also hypothesised that in the pre-group sessions, validating adolescents’ experiences would elicit hope and lead to early improvements in depression.

Ahead of the phase III trial, we refined these initial CMOCs based on qualitative and quantitative analyses from the pilot cRCT (manuscript in preparation). Briefly, qualitative data inferred that whilst adolescents from lower socioeconomic backgrounds were engaged in IPT, those experiencing more extreme adversity were often unable to attend sessions and therefore had limited engagement. Regarding school climate, quantitative analyses suggested IPT was effective across different levels of school climate, potentially compensating for the effects of a negative school climate on adolescent mental health. Qualitative data suggested gendered mechanisms of IPT: for girls, IPT served as a platform to share their feelings and learn skills, whereas boys learned how to reduce conflict. We found no qualitative or quantitative evidence that hope mediated early improvements in depression. The refined CMOCs are described in the process evaluation section of this protocol.

### Aims and objectives

Through a phase III realist cRCT, we aim to evaluate the effectiveness and cost-utility of IPT for adolescents with depression in Nepal and to understand how IPT works, for whom and in what circumstances. Our objectives are to:

i. examine the effectiveness of IPT on depression (the primary outcome), and secondary outcomes using a cluster randomised controlled trial design;
ii. using qualitative and quantitative process data, test hypotheses (CMOCs) informed by the intervention theory of change about mechanisms and outcomes, and how they vary by contextual characteristics of place and person;
iii. conduct an economic evaluation combining cost-utility and benefit-cost analyses to assess costs and benefits accrued during intervention and follow-up, employing provider and modified societal perspectives;
iv. explore the implementation of IPT in terms of feasibility, fidelity, reach, dose and acceptability through a mixed methods process evaluation.

We also plan to collect qualitative and quantitative data from participants at 12 months, 18 months and 30 months post-randomisation This will enable us to conduct secondary analyses that examine how participation in IPT affects long term outcomes.

## Methods

This phase III realist cRCT will broadly follow the same protocol as the pilot cRCT but with changes to inclusion and exclusion criteria, data collection and implementation outlined in Table 1.

**Table 1:** Key changes to the phase III trial protocol informed by learning from the pilot trial

PROTOCOL CHANGE	JUSTIFICATION
Changes to inclusion and exclusion criteria	
Removal of functional impairment from inclusion criteria.	In the pilot cRCT, adolescents with depression were only included if they also reported functional impairment. This was in line with previous studies in Nepal which combined depression and functional impairment measures to optimise the sensitivity of screening procedures for depression [18, 51]. However, there has been a recent, rigorous validation of the PHQ-A in Nepal reporting sufficient sensitivity and selectivity for a cut off score of 11 or more on the PHQ-A [36]. These results negate the need for a measure of functional impairment at screening though we will still include functional impairment as a trial outcome.
Inclusion of adolescents in grade 7	Previously we did not include adolescents in grade 7 because they were perceived as insufficiently mature to benefit from IPT. However, because adolescents frequently have to repeat grades when they fail exams, many older adolescents are studying in grade 7. Therefore, it is more appropriate to exclude adolescents on the basis of age rather than grade, so we will extend screening to grade 7.
Exclusion of adolescents in grade 11	In the phase III trial, we will not include adolescents in grade 11 because the number of those adolescents is comparatively low, making it difficult to form IPT groups with sufficient numbers. Moreover, it was difficult to combine adolescents from grades 8, 9 and 11 in groups because grade 11 adolescents had different timetables and did not appear to bond with younger group members.
Removal of c*hhopne* from exclusion criteria	Adolescents were excluded from the pilot trial if they screened positive for *chhopne* (local term for symptoms in line with conversion disorder) [29]) in the past three months because of concerns about whether IPT would be an appropriate treatment. Based on our experience in the pilot cRCT, we would prefer to include these adolescents and provide facilitator training and guidance in the manual on how to manage their symptoms.
Revision of exclusion criteria related to suicidal behaviour	In the pilot cRCT, we assessed suicidality using the Columbia Suicide Severity Rating Scale (CSSRS) and excluded adolescents with suicide intention or plan in the last two weeks or attempt within the last three months [30]. However, during the pilot cRCT, the team raised concerns about the specificity of the CSSRS, specifically that we were excluding adolescents who were not at high risk and could have benefitted from IPT. Consequently, we have undertaken extensive formative work to revise the CSSRS translation and our risk definitions, including interviews with adolescents and Nepali clinical experts. We will exclude high-risk adolescents defined as those with (i) suicide plan in the past 2 weeks, ii) suicide attempt in the past three months or iii) lifetime suicide attempt with suicidal ideation in the past two weeks.
Changes to data collection	
Timing the surveys relative to randomisation	We timed midline, endline and follow-up surveys in the pilot cRCT according to how far the intervention arm had progressed through the intervention (i.e. based on group session). This was difficult to operationalise because IPT groups progressed at different speeds due to school holidays, exams and absences, and it was not obvious when to conduct surveys with adolescents in the control arm. In the phase III trial, all surveys will be conducted based on time since randomisation (designated week 0).
Removal of second midline survey	We will not conduct a second midline survey in the phase III trial because we do not think the scientific insight from the survey justifies the resources required and burden imposed on adolescents and schools. Furthermore, the purpose of the midline survey is to identify the mechanism through which IPT improves depression (i.e., indirect effects). Data from the pilot trial revealed that the indirect effects were similar across the two midline surveys (week 3 and week 6).
Surveys with mothers and fathers	In the pilot cRCT we assessed the feasibility of conducting surveys with a subgroup of adolescents’ mothers. We were able to collect data from 73% of mothers at baseline (40/55) including data on socioeconomic status and mental health. This suggests that it is feasible to conduct surveys with mothers in the phase III trial, but to improve the participation rate and reduce missing data we will conduct surveys with adolescents’ mother or father – whomever is available to participate. This will enable us to test hypotheses concerning the influence of parents’ socioeconomic status and mental health on adolescent trial outcomes.
Changes to implementation	
Strategies to optimise attendance at IPT sessions	We narrowly met progression criteria for attendance at IPT sessions in the pilot cRCT. To improve attendance in the phase III trial, we will introduce the following measures: • Optimising the timing of the intervention – we now have a detailed calendar of school holidays, closures and exam periods and therefore optimised the timing of the intervention to avoid these as much as possible. • Excluding grade 11 – in the pilot cRCT we combined grades 8/9 and 11 in some groups. Because of differences in class timetables for these grades we had few viable timeslots for IPT sessions. Timetables for grades 7, 8 and 9 have similar free periods and more mutually convenient timeslots, which will improve attendance. • Guidance on managing absenteeism in the IPT manual – this includes scripted conversation and motivational-interviewing techniques. • Showing a film to staff and students in intervention schools as part of an orientation session – we made a short film about IPT involving participants from the pilot trial to psychoeducate and destigmatise participation in IPT groups.

### Study design

The trial is being implemented by Transcultural Psychosocial Organization Nepal (TPO Nepal) in partnership with University College London (UCL). We will conduct a superiority phase III cRCT to test whether IPT is more effective at reducing symptoms of adolescent depression compared to enhanced usual care. Schools are the unit of allocation and the trial will comprise 48 schools (24 intervention, 24 control).

Schools will be divided into eight groups or *waves* (six schools per wave). Advantages of grouping schools into eight waves include: (i) the duration between identifying that an adolescent is depressed at screening and the adolescent starting IPT is minimised; (ii) a staggered start to the surveys - meaning fewer research assistants and IPT facilitators are needed over the course of the trial; and (iii) randomisation can be conducted after the baseline survey with minimal delay. Schools will be allocated to each wave based on their location so that schools in the same area are allocated to the same wave. This will make it easier for the research and clinical teams to travel from school to school to provide supervision and oversight. Schools in each wave will be randomly allocated 1:1 to intervention/control conditions.

Within each wave, we will assess primary and secondary outcomes at baseline (pre-randomisation), endline (17 weeks’ post randomisation) and follow up (32 weeks’ post randomisation). We will also conduct a midline survey to assess intermediate outcomes five weeks after randomisation. The primary analysis will be a cross-sectional comparison of mean depression symptom scores at endline across trial arms, adjusted for baseline scores and clustering. Longer term follow-up will be conducted at 12-, 18- and 30-months post randomisation respectively.

### Setting

The trial will be conducted in schools in Chitwan and Nawalpur districts in the lowland region of Nepal. Both districts are mainly rural, though Bharatpur metropolitan city in Chitwan is the third most populous city in Nepal (~ 369k residents). In The 2021 national census, Chitwan had a population of 719,859 and 17.9% were adolescents aged 10–19 [31]. Nawalpur had a population of 378,079 and 18.8% were adolescents. The percentage of the population aged 5 and over who could read and write was 83.7% in Chitwan and 82.4% in Nawalpur, higher than the national average of 76.2%. The most populous ethnic group in Chitwan is Tharu and in Nawalpur is Magar [31].

IPT will be delivered in public secondary schools. Most adolescents in Nepal attend public schools. Around 84% of school children are in public schools at basic education level (grades 1–8), 81% at secondary level (grades 9–10) and 71% at higher secondary level (grades 11–12) [32]. Public education in Nepal is free until grade 10, though schools may charge registration fees and require students to purchase uniform and stationery [33].

In terms of mental health care in the study setting, there are several non-governmental organisations offering psychosocial counselling. Hospitals in urban areas provide psychiatric outpatient and in-patient services and some also offer counselling services. However, there has been limited research on adolescent mental health in this setting. A school-based study in Chitwan reported 27% prevalence of depression (using a PHQ-9 cut off score of > = 10) among 371 adolescents aged 15–19 [34].

### Study population

The study population is adolescents with depression aged 13–19 attending a school selected for the trial. Adolescents commonly repeat school years due to failing their end of year exams. Because of this, the age range in each grade is wide. We will focus recruitment on grades 8 and 9 but if there are insufficient adolescents with depression in these two grades (i.e. less than five boys or girls), we will recruit from grade 7. This age group is targeted because: depression often presents first in adolescence with a peak age of onset of 20.5 [35]; data from the pilot trial and feasibility study in Nepal indicated that IPT is feasible and acceptable for this age group; it is feasible to work with adolescents in grades 7 to 9 because they have more free time and attend school regularly compared to students in higher grades; and evidence from other settings suggests IPT reduces depression for this age group.

### Inclusion and exclusion criteria

Eligible schools are secondary schools within the public education system in Chitwan (Bharatpur Metropolitan City) and Nawalpur (Gaidakot and Devchuli municipalities). We will exclude private schools and single sex schools, as well as any schools in which we have previously run IPT groups as part of facilitator training. We will also exclude small schools defined as those with fewer than 100 students in grades 7, 8 and 9 combined, based on the most recent enrolment data available. This is because, based on an estimated 70% of adolescents returning signed consent forms and a 15% eligibility rate with a minimum of five adolescents per group, we are unlikely to be able to identify enough adolescents to form boys’ and girls’ IPT groups in these schools. We will also exclude one exceptionally large school (> 850 students) due to logistical challenges of screening many adolescents.

Within selected schools, eligible participants will be adolescent boys and girls aged 13–19 enrolled in Class 7, 8 or 9, attending a participating secondary school and with depression (i.e. scoring 11 or more on the PHQ-A). Adolescents will be excluded if they are at high risk of suicide defined as (i) suicide plan in the past 2 weeks, ii) suicide attempt in the past three months or iii) lifetime suicide attempt with suicidal ideation in the past 2 weeks.

### Recruitment

We will approach schools initially by phone call and arrange to meet with the principal to explain the study and obtain approval. The team will then run an orientation session for teachers. Research assistants will visit classrooms to present the study to adolescents. All adolescents in the classrooms will be given a consent form and instructed to bring the consent form back (signed by themselves and a caregiver) if they are interested in being screened and potentially participating in the trial. Each signed consent form will be assigned an ID number. ID numbers will be randomly ordered using an online platform (e.g., https://www.random.org/lists/). The research assistants will invite adolescents for screening according to this random order and screen them for eligibility.

Based on our experience in the pilot cRCT, the optimum number of adolescents in an IPT group is eight, with a minimum of five. Based on depression prevalence estimates from our pilot cRCT, recruiting eight boys and eight girls from grades 8 and 9 would be feasible in large schools. However, in small schools we expect that even five might be challenging and in that case we may need to screen grade 7 students who fall within our target age range (13–19). Our experience suggests it is preferable to have smaller groups of adolescents from grades 8 and 9 than to have larger groups of adolescents from grades 7, 8 and 9. This is because facilitators in the pilot trial reported that groups combining students from grades 7, 8 and 9 tended to be less cohesive than groups with students from grades 8 and 9 only. Therefore, we will screen grade 7 only when it is necessary to form a group according to the following recruitment procedure:

In all schools we will initiate screening parallelly in grades 8 and 9 and continue until either:

(i) we recruit eight boys and eight girls (plausible in larger schools); or.
(ii) we have screened all adolescents in grades 8 and 9.

If (ii) occurs and we have recruited fewer than five boys and/or five girls, we will initiate screening in grade 7 and continue until we have recruited eight boys and/or eight girls in total across the three grades, or until we have screened all adolescents in grade 7.

Those adolescents meeting the eligibility criteria will complete the baseline survey and excluded participants will be informed at the end of the screening by the research assistant. Adolescents who are excluded due to suicidality will be referred for counsellor assessment.

### Randomisation

Randomisation will be at the school level and conducted for each wave (i.e. on eight separate occasions) by two independent statisticians. In each wave, once screening is complete one statistician will generate a randomised list of numbers 1–6 allocated in a 1:1 ratio across intervention and control arms. Independently and in parallel, the second statistician will randomise the order of the six school names. The Principal Investigator (PI) will then apply the school names in the order received from the second statistician to the allocations provided by the first statistician. The PI will send the final allocation to the two statisticians to approve. Separating randomisation and allocation tasks in this way will ensure that neither the statisticians nor the PI can manipulate the assignment of schools to trial arms.

## Intervention and comparator

### Intervention

We will evaluate the IPT intervention implemented in the feasibility study and pilot cRCT. The intervention involves two pre-group sessions and 10 group sessions. Sessions generally last 90 to 120 min and are held weekly unless there is a need to alter the schedule due to e.g. school closures or participant availability. In the first pre-group session, the facilitator meets the participant one to one to identify the most relevant IPT problem area, help the participant link their depressive symptoms to the problem area, and gather information about the participant’s key relationships and history of depression. In the second pre-group session, the facilitator meets the participant and their caregiver together, ideally at home, to mobilise support and build rapport with the participant’s family. Where a participant’s depression is related to their family circumstances, the facilitator will discuss with the participant in advance about what they are comfortable to share with their caregiver.

IPT groups are gender specific and comprise 5–8 participants per group. Each group has two facilitators. Group sessions take place in a quiet, private space in the school (such as an empty classroom or the library). In the initial group session, the facilitator focuses on encouraging participants to review and share their problems, and instilling hope for recovery. In the middle sessions (2–9), participants practice interpersonal skills and offer and receive support from group members to resolve their problems. In the last session, participants review and celebrate progress and make plans to tackle future problems.

In each pre-group and group session, participants review their depressive symptoms with the facilitator using a nine-item symptom checklist developed specifically for the study. This review process helps participants link changes in symptoms to events in their daily lives and enables facilitators to identify deterioration and suicidality.

We will recruit lay people to train as IPT facilitators. The project clinical coordinator and supervisors who are trained in IPT will conduct the training. The training programme comprises three modules: (i) WHO’s *Foundational Helping Skills* – a 10-day module to build basic psychosocial skills (ii) a one-day module focused on group management; and (iii) an IPT module involving an eight-day didactic workshop focused on theory, structure, techniques and strategies, followed by supervision of a minimum of three practice cases including two individuals and one practice group of four or five adolescents.

At each stage of the training, we will assess facilitators’ competency. Foundational helping skills will be assessed during standardised role-plays pre- and post-training using the Enhancing Assessment of Common Therapeutic factors (ENACT) rating scale [36]. We will assess group management skills in role plays and practice groups using GroupACT [37]. Facilitators’ understanding of the IPT model will be assessed after the didactic workshop using a paper-based knowledge test. During practice groups, supervisors will assess IPT skills using a standardised rating scale of activities carried out in each session. Based on competency and availability, we will select the trained facilitators for the trial.

### Comparator

Participants attending schools in the control arm will receive enhanced usual care. This is justified in terms of balancing the generalisability of our findings and ethical obligations as researchers. In intervention and control arms, we will train health workers in health posts and primary care centres using the WHO mental health Gap Action Programme (mhGAP) intervention guide. Participants in control and intervention clusters will receive information about the location of these trained health workers and how they can access treatment.

During the trial in both arms, participants who report a suicide plan in the past two weeks, a suicide attempt in the past three months or a lifetime suicide attempt and ideation in the past two weeks will be assessed by a psychosocial counsellor employed through the project and referred to mental health services as per need. These participants will not be withdrawn from the outcome assessment. If the participant is in the intervention arm they will continue with IPT if they wish to and the clinical team deems it appropriate.

To incentivise participation among schools in intervention and control arms, in each wave after endline, we will invite one or two teachers per school to participate in a psychoeducation workshop.

### Trial outcomes and measurement

Table 2 details the trial outcomes and tools to measure them. The primary outcome is depression symptoms at endline measured as a continuous outcome. We will measure this using the PHQ-A [38]. This tool is widely used across cultures and contexts, has been adapted for and validated among adolescents in Nepal and is sensitive to change [39].

**Table 2:** Trial outcomes and tools

Outcome type	Outcome	Tool/measure	Assessment timepoint
Primary	Depression	Patient Health Questionnaire modified for adolescents (PHQ-A)	Baseline, midline, endline, follow-up
Secondary	Functional impairment	Locally developed tool	Baseline, midline, endline, follow-up
Secondary	Anxiety	Generalised Anxiety Disorder-7 (GAD-7)	Baseline, midline, endline, follow-up
Secondary	Post-traumatic stress disorder	PTSD Checklist for DSM-5 (PCL) 8-item	Baseline, midline, endline, follow-up
Secondary	School attendance	No. of days attended in 24 days prior to baseline (excluding closures and Saturdays)	Baseline, midline, endline, follow-up
Secondary	Health-related quality of life	EuroQol-5 Dimension 5 levels (EQ-5D)	Baseline, midline, endline, follow-up
Secondary	Academic self-efficacy	Young Lives Non-Cognitive Scales	Baseline, midline, endline, follow-up
Secondary (long term)	Academic performance	Basic Education Examination School Education Examination grade	Follow-up
Secondary (long term)	School drop out	Self-report and school register	Endline and follow-up
Intermediate	Hope	Herth Hope Index	Baseline, midline, endline, follow-up
Intermediate	Emotion regulation	Difficulties in Emotion Regulation Scale (DERS-SF)	Baseline, midline, endline, follow-up
Intermediate	Self-efficacy	General Self-Efficacy Scale	Baseline, midline, endline, follow-up
Intermediate	Social support	Multidimensional Scale of Perceived Social Support (MSPSS)	Baseline, midline, endline, follow-up
Intermediate	Group cohesiveness	PM + Group Cohesiveness scale adapted for IPT	(Intervention arm only) midline, endline, follow-up
Intermediate	Interpersonal conflict	Network of Relationships Questionnaire	Baseline, midline, endline, follow-up
Predictor/moderator	School climate	Beyond Blues School Climate Scale	Baseline
Predictor/moderator	Socioeconomic status	Social and Economic Measure including household assets and food security	Baseline
Predictor/moderator	Parent mental health	General Health Questionnaire	Baseline, endline
Predictor/moderator	Parenting behaviour	Alabama Parenting Questionnaire	Baseline, endline
Predictor/moderator	Disruptive behaviour of adolescents – parent report	Disruptive Behavior International Scale – Nepal version (DBIS-N)	Baseline, endline
Predictor/moderator	Gender norms	Johns Hopkins Global Early Adolescent Study Measure	Baseline
Predictor/moderator	Adversity	Johns Hopkins Global Early Adolescent Study Measure	Baseline

We will assess other potential intervention effects by measuring the following secondary outcomes: anxiety symptoms measured with the Generalised Anxiety Disorder-7 (GAD-7) validated in Nepal [39, 40]; functional impairment using a tool developed specifically for the study setting based on methods outlined in Bolton et al. [41]; PTSD symptoms with the 8-item version of the PTSD Checklist for DSM-5 (PCL-5) [42] based on longer versions previously used in Nepal [39, 43–45]; and quality of life with the EuroQol-5 Dimension 5 levels (EQ-5D) [46]. Other secondary outcomes are school attendance (number of days attended in 24 days prior to the survey excluding closures and Saturdays) and academic self-efficacy using questions from the Young Lives non-cognitive instrument, translated and tested in Nepali [47]. Academic self-efficacy is a construct associated with goal setting and academic strategy use and conceivably lies on the pathway to improved academic performance [48]. In follow-up surveys post-intervention, we will examine long-term effects of IPT on school drop-out and performance in two standardised examinations, the Basic Education Examination (BEE) and Secondary Education Examination (SEE).

Informed by our theory of change, we will assess intervention effects at midline, endline and follow-up on intermediate outcomes and whether these outcomes mediate the effect of the intervention on depression. Intermediate outcomes are: hope measured with the Herth Hope Index [49]; emotion regulation with the Difficulties in Emotion Regulation Scale (DERS) [50]; self-efficacy with the Generalised Self-Efficacy Scale [51]; social support with the Multidimensional Scale of Perceived Social Support [52]; and interpersonal conflict reduction using select items from the Network of Relationships Questionnaire [53]. In the intervention arm, we will measure group cohesion using a tool developed to assess cohesion in a trial of Problem Management Plus in Nepal [54], which we adapted for IPT and used in the pilot trial.

### Assessment and follow up

We will conduct baseline surveys with adolescents before randomisation and collect data on demographic characteristics, primary and secondary outcomes and intermediate outcomes. We will also collect data on potential predictors and moderators of intervention effects including school climate, gender norms, adversity and socioeconomic status. We will measure school climate with an abbreviated version of the Beyond Blues School Climate Scale [55] and gender norms and adversity using select items from the Johns Hopkins Global Early Adolescent Study [56]. We will collect data on indicators of wealth including household assets and food security to generate a composite indicator of socioeconomic status. We will resurvey adolescents at midline, endline and follow-up, and for longer term follow up assessments at 12-, 18- and 30-months post randomisation. Based on the pilot cRCT, we expect a 92% response rate at endline.

We will also conduct surveys at baseline with adolescents’ parents (mother or father) to collect data on potential moderators of intervention effects. We will measure parents’ mental health using the General Health Questionnaire 12-item [57], parenting behaviour with the Alabama Parenting Behaviour Scale (involvement (10 items), positive parenting (6 items) and corporal punishment (3 items) subscales) [58] and disruptive behaviour with the Disruptive Behaviour International Scale – parent report [59]. We will survey parents again at endline to collect data for an economic analysis. If the adolescent does not have a parent or the parent is unavailable for interview, this will be recorded and linked to the adolescent’s data.

All surveys will be conducted by trained research assistants using tablets programmed with data collection software. Research assistants will read the survey questions to the participant and enter their responses on the tablet. We will conduct as many surveys as possible in person. Where we cannot meet the participant in person, we will conduct the interview on the phone. In-person surveys will be conducted in a private place at the participant’s school (adolescents) or home (adolescents and parents). For phone surveys, we will arrange a time for the survey in advance. Research assistants but not participants will be masked to allocation.

### Process evaluation data collection

The process evaluation design is informed by frameworks and theory including the general theory of implementation and Medical Research Council guidance [60, 61]. Process data will be quantitative and qualitative. We will collect core process evaluation data (including fidelity and acceptability) across all intervention schools and from all IPT participants. We will select 16 case study schools (eight in the intervention arm and eight in control) for in depth qualitative process data collection. We will purposively sample schools across different waves, municipalities and urban and rural areas. We will include smaller and larger schools. The process evaluation will assess intervention implementation, mechanisms of change and contextual factors.

### Implementation

We will assess fidelity, reach and acceptability of the intervention across all sites and participants. We will examine fidelity to explore whether IPT was delivered as per the intervention manual and by competent facilitators. We will assess the percentage of IPT sessions delivered as per the manual, extracting this information from facilitator records. We will measure facilitator competency according to the assessment schedule detailed above. These data will be extracted from supervisor records. During IPT session observations, supervisors will use adherence checklists developed for each phase of IPT to assess the extent to which standardised components of IPT are being implemented and at what level (satisfactory, superior). Through focus group discussions with facilitators and supervisors we will explore deviations from the manual and whether they were intentional or otherwise, and whether they deviated or adhered to the theory of change. Within IPT, adolescents are encouraged to generate and select solutions to their interpersonal problems with the support of group members and facilitators. Through qualitative interviews with adolescents, focus group discussions with facilitators and supervisors, and analysis of IPT session recordings, we will explore whether these solutions were consistent with our theory of change. We will explore how fidelity varies across schools, IPT groups and over the course of the trial, and potential contextual factors that affect this.

Reach is defined as the extent to which the target population comes into contact with the intervention [60]. We will assess the percentage of schools and individual adolescents approached that agree to participate.

To assess acceptability, we will collect quantitative and qualitative data. In terms of quantitative data, across intervention schools, we will assess the percentage of IPT sessions attended by adolescents using information from facilitator attendance records (overall and by gender, caste/ethnicity and socioeconomic status). In all intervention schools, we will administer treatment satisfaction surveys to IPT participants in the last group session. In terms of qualitative data on acceptability, we will conduct interviews and focus group discussions with adolescents, caregivers and teachers in case study schools as well as facilitators and supervisors, using topic guides informed by the General Theory of Implementation [61]. We will explore: whether IPT is perceived as needed and valuable (coherence) and if this changes over the course of the intervention; how willing adolescents, families and schools were to invest in and commit to IPT (cognitive participation) and in what ways they demonstrated this commitment; how well IPT fitted into existing school and household routines (collective action); and how people felt IPT went (reflexive monitoring). We will also explore: intervention capability (whether IPT was workable within the education system); participant potential (individual and shared readiness for the intervention); institutional capacity (whether existing material and cognitive resources were sufficient, and how social norms and roles served to promote or undermine intervention activities); and contextual factors that affect intervention acceptability, for example whether implementation was better in schools that perceived a greater need and demonstrated more commitment to IPT.

To describe enhanced usual care in the control arm, we will examine quantitative data on adolescent mental health service use in surveys, review case notes for participants referred to the counsellor (number of participants assessed, number of counselling sessions conducted, number and type of referrals made) and conduct focus group discussions with the counsellors. Contamination across arms is extremely unlikely and only possible if for example, a participant in a control school and participant from the intervention arm were in the same household or friendship group, or a participant from the intervention arm moves to a school in the control arm. Through surveys with parents, we will identify if any participants live in the same household. From IPT facilitator notes, we will be informed if any intervention participants move school.

Table 3 details qualitative and quantitative data sources for the process evaluation.

**Table 3:** Qualitative and quantitative data sources for the process evaluation

Data source	Timing	No. of participants	No. of schools (intervention/control)	Evaluation domain addressed
Quantitative data				
Adolescent surveys	Baseline, midline, endline, follow up	480 to 768 (all participants)	*N* = 48 (Int: *N* = 24/Cont: *N* = 24)	Outcomes Contamination Mechanisms Context
Parent surveys	Baseline, endline	480 to 768 (parents of all participants)	*N* = 48 (Int: *N* = 24/Cont: *N* = 24)	Contamination Context
Facilitator competency assessments	Pre-training, post training, for each cycle and for each facilitator ENACT in pre-group sessions, GroupACT in 4 group sessions	All facilitators	N/A	Implementation
Session observations using IPT adherence checklist	Waves 1–8	All facilitators (~ 25% of total sessions)	*N* = 24 (I)	Implementation
School attendance records	Baseline, endline	480 to 768 (all participants)	*N* = 48 (Int: *N* = 24/Cont: *N* = 24)	Outcomes, implementation
IPT session attendance	Per each IPT session	240 to 384 (all intervention participants)	*N* = 24 (I)	Implementation
IPT treatment satisfaction survey	Endline	240 to 384 (all intervention participants)	*N* = 24 (I)	Implementation
Group cohesiveness assessment	Midline, endline	240 to 384 (all intervention participants)	*N* = 24 (I)	Implementation, mechanisms
Facilitator session and supervision records	Per each IPT session	All facilitators	N/A	Implementation, context
Counsellor notes from high risk and abuse-related cases	Per each case referred to the counsellors	All counsellors	N/A	Implementation
Qualitative data				
Audio recordings of sessions	(Supervisor records sessions observed) For each cycle: for each facilitator, rating two individual sessions and 4 group sessions	N/A	*N* = 24 (I)	Implementation, mechanisms
Interviews with caregivers of adolescents participating in IPT	Endline	16 (2 per school)	*N* = 6 (Intervention case study schools)	Implementation, context, mechanisms
Interviews with adolescents participating in IPT	Endline	32 (4 per school)	*N* = 6 (Intervention case study schools)	Implementation, context, mechanisms
Interviews with adolescents in the control arm	Endline in case study schools (Cycles 1–4)	8 (1 per control case study school)	*N* = 8 control case study schools	
Focus group discussions with facilitators and supervisors	Endline (Wave 4 and 8)	2–3 FGDs in Wave 4; 2–3 in Wave 8, 4–6 facilitators/supervisors per FGD	N/A	Implementation, context, mechanisms
Focus group discussions with counsellors and supervisors managing high-risk cases	Endline (Wave 2 and 6)	All counsellors and supervisors, 4–6 per FGD	N/A	Implementation
Teacher one to one interviews	Endline in case study schools	Principal (*N* = 16 I = 8/C = 8), teachers (*N* = 16 I = 8/C = 8)	*N* = 16 case study schools (Int: *N* = 8/Cont: *N* = 8)	Implementation, context
Interviews with caregivers of adolescents in the control arm	Endline in case study schools	8 (1 per control case study school)	N/A	Implementation

### Mechanisms of change and contextual factors

Informed by realist evaluation principles and work by Bonell and colleagues on realist randomised trial methodology [20, 21, 62], the process evaluation seeks to refine our theory of change about how IPT works, for whom and in what circumstances. To do this, we have generated hypotheses in the form of CMOCs about how intervention mechanisms and contextual factors interact to generate outcomes. We define mechanisms as changes in the reasoning and behaviour of individuals or the way in which institutions operate in response to local use of intervention resources [21]. Whilst mechanisms cannot be directly observed they can be inferred for example through quantitative data on mediator variables or qualitative data collected from intervention participants. We define context as aspects of the place or people within which an intervention is implemented that might affect how the mechanisms operate. Context can relate to material or non-material features (social, psychological, organisational, political and economic), and operate at all levels of the social ecological framework including individual, family, school, community and government levels.

We also propose ‘dark logic’ hypotheses related to mechanisms of potential harm, where mechanisms of harm might generate ‘paradoxical effects’ on our primary or secondary outcomes or generate ‘harmful externalities’ in other domains [63].

We will draw on findings from analyses of the qualitative process data to refine (including removing or adding to) our hypotheses. This ensures we remain open to pathways that are not already specified in our process evaluation. We will then test these refined hypotheses using analyses drawing on quantitative trial data to check these data align with our theoretical predictions [62]. We will use mediation analyses to examine underlying mechanisms that explain the effect of IPT on adolescent depression. Moderation analysis will be used to assess whether a measure of contextual factor moderates the effect of IPT on depression. We will also use moderated mediation analysis to test whether mediation is contingent on a contextual factor.

We use categories proposed by Bonell and colleagues in a previous realist RCT to organise our hypotheses [64]. These categories are intervention mediators, contextual factors affecting implementation, contextual factors modifying intervention effects and potential harms. Table 4 presents our CMOCs and indicates the data and analyses we will use to test them.

**Table 4:** Process evaluation hypotheses and associated data and analyses

Hypotheses	Qualitative data sources/analyses	Quantitative data sources/analyses
Hypotheses about intervention mediators		
H1: Adolescents participating in IPT will report increased social support at midline, endline and follow up due to bonding between group members, initiation of new relationships outside groups and/or improvement in the quality of existing relationships. This generates reductions in depression.	Analysis of SSI transcripts with adolescents and parents and FGD transcripts with facilitators from different groups (direct and indirect probing on hypothesis)	Mediation analysis to assess if IPT affects PHQ-A outcomes at endline through social support (MSPSS score at midline)
H2: Adolescents participating in IPT will report reduced interpersonal conflict at midline, endline and follow-up due to improved communication of interpersonal needs and strategies to better manage anger. This generates reductions in depression.	Analysis of SSI and FGD transcripts with adolescents and facilitators from different groups (direct and indirect probing on hypothesis)	Mediation analysis to assess if IPT affects PHQ-A outcomes at endline through interpersonal conflict (Social Adjustment Scale Self Report score at midline)
H3: Adolescents participating in IPT will experience improved emotion regulation at midline, endline and follow up because they learn how to name and express their emotions. This generates reductions in depression.	Analysis of SSI transcripts with adolescents and parents and FGD transcripts with facilitators from different groups (direct and indirect probing on hypothesis)	Mediation analysis of adolescent survey data (DERS-SF and PHQ-A outcomes)
H4: Adolescents participating in IPT will report increased hope at midline as participants perceive the possibility of change in mood and relationships. This generates reductions in depression.	Analysis of SSI transcripts with adolescents	Mediation analysis of adolescent survey data (Herth Hope Index and PHQ-A data)
H5: Adolescents participating in IPT will report improved self-efficacy at midline, endline and follow-up because they experience generating solutions to and solving their interpersonal problems. This generates reductions in depression.	Analysis of SSI transcripts with adolescents and FGD transcripts with facilitators from different groups	Mediation analysis of adolescent survey data to assess if self-efficacy mediates PHQ-A.
H6: Adolescents participating in IPT will report improved self-compassion at midline, endline and follow up because in IPT groups they normalise and validate their experience of depression. This generates reductions in depression.	Analysis of SSI transcripts with adolescents and FGD transcripts with facilitators from different groups	We will not have quantitative data to test this hypothesis.
Hypotheses about contextual factors affecting implementation		
H7: Adolescents participating in IPT who are from the poorest backgrounds whose basic needs are not met will attend fewer IPT sessions because they have to undertake paid work and/or have household responsibilities to fulfil.	Analysis of SSI transcripts with adolescent and parents and FGD transcripts with facilitators from different groups (direct and indirect probing on hypothesis)	Analysis of adolescent IPT session attendance data and socio-demographic data from baseline survey
H8: Groups involving adolescents across two grades (8/9) rather than three (7/8/9) will be more cohesive and attendance among participants will be higher because participants will be closer in age and maturity and sessions will be easier to schedule across fewer timetables.	Analysis of FGD transcripts with facilitators and SSIs transcripts with teachers (direct and indirect probing on hypothesis)	Analysis of group cohesion and adolescent IPT session attendance data.
H9: In schools that have experienced a suicide, an attempted suicide or mass conversion disorder (chopnne), IPT will be more acceptable and implemented with more fidelity because staff will be more motivated to support the programme.	Analysis of SSI transcripts with teachers and principals, FGD transcripts with facilitators	We will not have quantitative data to test this hypothesis.
Hypotheses about contextual factors influencing intervention effects (CMOCs)		
H10: Adolescents participating in IPT who attend schools with a climate characterised by a lack of understanding and supportive teacher and student relationships (context), will build strong peer to peer social support, a group identity and a sense of belonging with IPT group members (mechanism) that will offset the negative effect of the school climate on adolescents’ depression (outcome).	Analysis of SSI transcripts with adolescents sampled to represent different school climates (baseline Beyond Blue score), teachers and principals, and FGDs with facilitators, probing on hypothesis	Comparison of group cohesion data across schools Moderator analysis of adolescent survey data (Beyond Blues School Climate Scale as moderator variable, PHQ-A outcome)
H11: Adolescents participating in IPT from a lower socioeconomic background (poorer household, context) will be more engaged and motivated to address their problems and have more problems to solve compared to their peers from wealthier backgrounds. These adolescents will share their feelings and problems and participate in discussions through which they learn strategies to regulate their emotions, reduce conflict, build social support and improve self-efficacy (mechanism) and therefore experience more benefit from IPT compared to peers from a higher socioeconomic background (outcome).	Analysis of SSI transcripts with adolescents from different socioeconomic backgrounds and facilitator FGD transcripts (direct and indirect probing on hypothesis)	Moderator analysis of adolescent survey data (socioeconomic moderator variable and PHQ-A outcome)
H12: For girls, who are subject to gender norms that discourage them from expressing their feelings (context), group IPT will serve as a platform to become aware of and share feelings and learn emotion regulation skills (mechanism) which reduce their depression. On the other hand, boys who are exposed to gender norms which promote aggression and anger (context), through IPT learn how to reduce interpersonal conflict (mechanism) and therefore experience reductions in depression (outcome).	Analysis of SSI transcripts with boys and girls, and facilitator FGD transcripts (direct and indirect probing on hypothesis)	Moderated mediation analysis of adolescent survey data (gender moderator, emotion regulation and conflict mediator variables, PHQ-A outcome)
H13: For adolescents with a parent who has a mental health problem (context), through IPT they will increase their social support, normalise and validate their feelings, generate and implement solutions to their problems and reduce conflict at home (mechanism). This will offset the effect of the parent’s mental health on the adolescent’s depression (outcome).	Analysis of SSI transcripts with adolescent and parents with and without mental health problems (GHQ-12 score at baseline) and FGD transcripts with facilitators from different groups (direct and indirect probing on hypothesis)	Moderator analysis to assess if parent GHQ-12 score at baseline moderates effect of IPT on PHQ-A score at endline
Hypotheses about potential harms		
H14: For adolescents with a background of trauma (context), who recount their traumatic experiences during group sessions and to whose experiences other group members cannot relate, IPT will exacerbate their distress due to re-experiencing the stressful event (mechanism), which will result in a worsening of depression (outcome).	Analysis of SSI transcripts with adolescents with and without trauma (baseline PCL score) and FGD transcripts with facilitators from different groups (direct and indirect probing on hypothesis)	Analysis of adolescent survey data (PCL, PHQ-A outcomes)
H15: Adolescents experiencing stigma from school and community members because of their participation in IPT (context) would attend fewer sessions and the stigma would cause them distress and their depression worsens.	Analysis of SSI transcripts with adolescents who have experienced stigma and facilitator FGD transcripts	Cross-sectional analysis of adolescent survey data (endline, questions about experience of stigma due to participation in IPT, PHQ-A
H16: In groups adolescents are encouraged to generate and select solutions to solve their interpersonal problems. If the group is poorly facilitated these solutions may run counter to our theory of change and could cause adolescents harm for example by inciting violence in the context of a dispute. This could exacerbate adolescents’ depressive symptoms.	Analysis of SSI transcripts with adolescents and facilitator FGD transcripts probing on the nature of solutions generated within groups and their consequences Analysis of information from facilitator session and supervision notes on solutions and consequences	We will not have quantitative data to test this hypothesis.
H17: Group members or facilitators may breach an adolescent’s confidentiality by disclosing sensitive information about them to individuals outside the group. This would be distressing for the adolescent and could have harmful consequences resulting in the worsening of their depression.	Analysis of SSI transcripts with adolescents and facilitator FGD transcripts probing for evidence of a breach in confidentiality	We will not have quantitative data to test this hypothesis.

### Economic evaluation data collection

We will primarily adopt a provider perspective for the economic evaluation; secondarily, we will adopt a modified societal perspective to additionally account for the out-of-pocket costs and opportunity costs to adolescents and caregivers related to adolescents’ healthcare utilisation. To estimate the costs of the intervention and control conditions incurred by the implementing agency, monthly project budget actuals will be entered in an Excel tool by TPO Nepal staff members and continuously updated throughout the trial. We will divide costs into start-up or implementation costs and allocate them to different cost centres and intervention activities by trial arm. Major expected cost categories for both intervention and control groups include *personnel*,* equipment*,* travel*,* supervision and monitoring*, and *finance and administration.* TPO Nepal staff members will allocate staff salaries between trial arms; identify relevant costs incurred during the pilot which also contribute to the full trial cost; and document use of facilities and supplies in both Chitwan and Kathmandu office locations.

To estimate indirect costs for the modified societal perspective, we will document productivity loss and informal care costs by recording adolescents’ time spent on intervention-related activities (including travel and participation) if relevant, as well as time and costs associated with outside healthcare utilisation. At endline and follow-up, we will collect data on adolescent health care visits since the baseline survey, including the time and out-of-pocket expense for each visit (across a range of non-school based formal and informal healthcare types available locally). For each type of healthcare service received, we will establish whether its cost is fully covered by out-of-pocket expenditures, or whether it was partially or fully subsidised by the provider. We will record the estimated subsidised cost of services using publicly available information; if subsidised costs are not available, we will contact service providers to establish appropriate estimates or ranges which will be tested in sensitivity analyses. We will collect productivity and informal care costs from interviews with all available parents at endline and follow-up, documenting the time parents have spent providing care as well as opportunity costs of care-seeking for both parents and adolescents. Adolescents will be asked about any earnings from paid work and opportunity costs of healthcare utilisation at midline, endline, and all follow-up surveys. Intervention participants will also be asked about opportunity costs of IPT attendance at midline and endline during group cohesion assessments to maintain masking of group allocation. Cost data will be collected prospectively throughout the trial in Nepali rupees (NPR) and reported in 2024 prices.

The primary health outcome of interest for the economic evaluation analyses is the quality-adjusted life year (QALY). QALYs will be calculated using EQ-5D-5 L health utility scores from adolescents at each planned timepoint for data collection. Using this data, we will derive QALY weights using existing value sets from India, which represent the closest cultural context for which EQ-5D-5 L value sets are available [65].

### Power and sample size

#### Primary analysis

The aim of the primary analysis is to test the overall impact of IPT on adolescent depressive symptoms compared to enhanced usual care through a two-armed cluster RCT with secondary schools as the unit of randomisation. To fulfil the study objectives, we plan to recruit 48 schools, with 24 schools per arm with 10 to 16 adolescents per school taking part, giving a range of 480 to 768 adolescent individuals taking part. As the sample size is fixed due to logistics, we will take the approach of estimating the effect which can be detected by our cluster randomised design. For the purpose of sample size calculation, we will need the intraclass correlation coefficient (ICC, the proportion of variation due to variation between clusters), the standard deviation of the depression measure, the arithmetic mean of the cluster size, the standard deviation of cluster size variation in order to calculate the coefficient of variation for cluster size (cluster SD/cluster mean), the number of clusters, desired power, alpha level, direction of test (here two-way) and the rate of dropout of schools and adolescents. This information will allow us to use clustersampsi, a stata module for cluster randomised trial power analysis.

We use the baseline standard deviation of included adolescents from the pilot trial PHQ-A, which is 3.05. Assuming the distribution of clusters is uniform across the 10 to 16 range, with an arithmetic mean of 13 and standard deviation of 1.97, results in a coefficient of variation for cluster size of 0.151. However, in the pilot study 12/161 (8%) of adolescents dropped out by endline (the primary endpoint), which would approximate one adolescent per cluster. Here we’ll assume both 8% and a more conservative drop-out of two per cluster giving an additional missingness rate at individual level of 15%. No schools dropped out in the pilot study, but we will also assume one school in each group will be lost to follow-up. This leaves us with 46 schools with an average of 12 or 11 per cluster and coefficients of variation of 0.16 and 0.181 respectively. We use both a conservative ICC of 0.07 for the PHQ-A based on data collected on symptoms of depression using the Depression Self Rating Scale during the IPT feasibility study [19], and a more liberal value of 0.03 as ranges from 0.05 to 0.01 are more typical in community interventions. Finally, we assume a two-way test with 90% power and alpha set to 0.05. Given these parameters the smallest detectable effect size at the primary outcome timepoint at 8% missingness and ICC = 0.07 would be 0.29 (0.3 at 15% missingness) and the design effect (the effective reduction in sample size) due to clustering 1.79 (1.73 at 15% missingness). For an ICC of 0.03, the smallest detectable effect size would be 0.25 at 8% missingness and 0.26 at 15% missingness (design effects DE of 1.34 and 1.31 respectively). Hence, the study is well powered to detect small to moderate effects. These standardized effect sizes correspond to group difference 0.9 or less on the PHQ-A scale.

#### Mediation analysis

Whilst well-powered to detect modest effect sizes of 0.29, the sample size also means that we should have power to assess the CMOCs with a selection of multi-level moderation, mediation and mediated moderation models. Nevertheless, more complex models and effects such as the mediated direct effect mean sensitivities of the tests will be lower and detectable effects will necessarily be larger. Estimates of power for mediation and moderation analyses will depend on the CMOCs selected for testing but here we give some guidelines. For mediation, taking a simplistic approach using Table 3 from Fritz and McKinnon 2007 [66], a sample size (assuming the loss to follow up rates above) of 46 schools of cluster size 12 or 11 and ICC of 0.07 gives effective sample sizes of 308 and 292 (or 412 and 386 for an ICC of 0.03) and will provide 80% power to detect small to moderate mediation effects with a and b path coefficients of > 0.26. The minimum sample size for this permutation of effects (minimum 148) allows a substantial buffer (50%) wherein the true sample size needed is larger due to the effects of clustering on the variance of the indirect effect being underestimated.

#### Moderation analysis

**Fig. 2:**
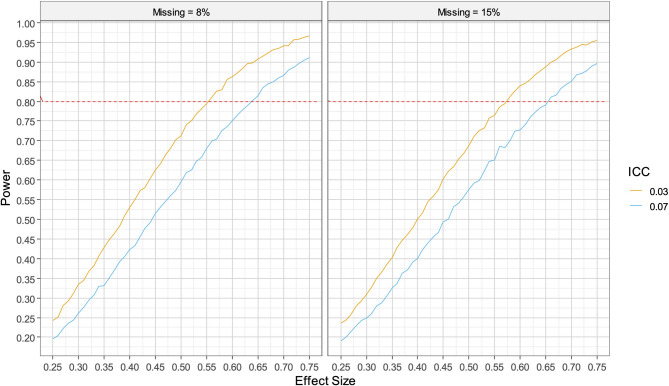
Power curves for moderation analyses

To evaluate power for a moderation analysis, we ran simulations at different effect sizes for our fixed sample size and design effects of 1.79, 1.73, 1.34 and 1.31 (depending on the ICC and coefficient of variation of group size at missingness rates of 8% and 15%) to address clustering and assuming alpha = 0.05. We used a simple model where treatment group interacted with a categorical variable such as gender dividing the treatment groups into equal sub-groups. The interaction effect size then represents the difference in the effect of treatment between the sub-groups and in the simulations ranged from 0.25 to 0.75 in increments of 0.01. As can be seen in Fig. 2, assuming a drop out rate of one school per group and 8% or 15% of participants in remaining schools, there will be power to detect a moderate to large effect size of 0.635 (ICC = 0.07, missingness = 8%, DE = 1.79), 0.655 (ICC = 0.07, missingness = 15%, DE = 1.73), 0.555 (ICC = 0.03, missingness = 8%, DE = 1.34) or 0.575 (ICC = 0.03, missingness = 23%, DE = 1.28). Given the baseline standard deviation of PHQ-A from the pilot trial (SD = 3.05) that gives a range of differences between groups of 1.7 to 2.0 on the PHQ-A scale.

#### Moderated mediation analysis for CMOCs

**Fig. 3:**
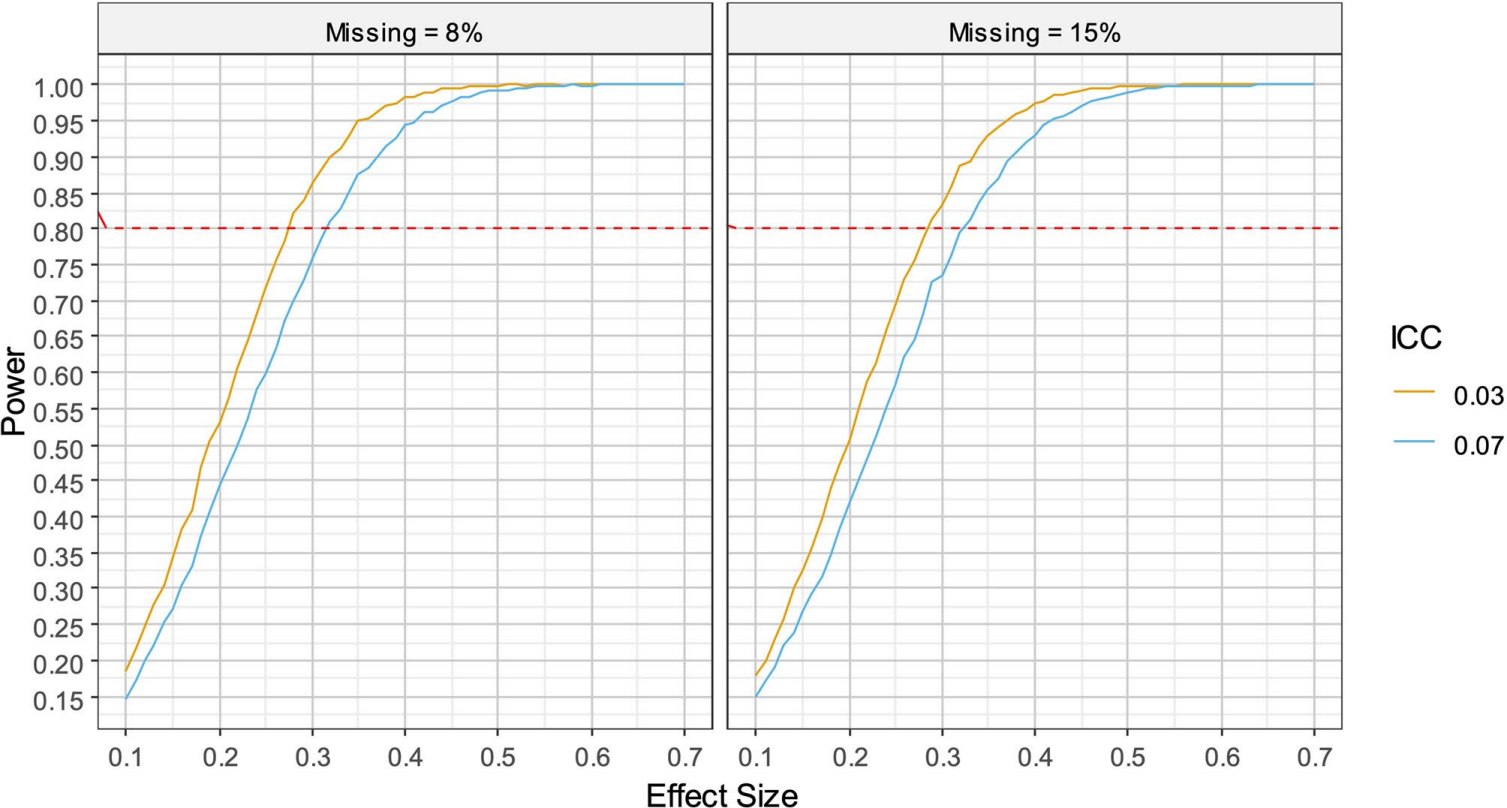
Power curves for the b path

In a moderated mediation, there are four variables: the independent variable; the outcome; the mediator; and a fourth variable which moderates the indirect path (either path a, path b or both). For illustration purposes, we use the case of a model in which the moderation happens to the a pathway only and assume gender moderates the relationship between treatment condition and the hope outcome. We use the above result for moderation as an estimate of the power for the moderated a pathway. Similarly, we can use a further simulation to find power of the b path, a model consisting of two predictors representing paths from the mediator and independent variable to the outcome. Assuming that the effects are independent, the power of the mediation or joint model is the power of the a path * power of the b path. If the value of this product is 0.8 or greater, then the a * b pathway will have 80% power. For example, this might occur if the power of the a path was 0.81 and the power of the b path 0.99 corresponding to a and b paths of at DE = 1.79 (the more conservative ICC estimate assuming missingness of 8% and 1 cluster per group). Other scenarios are shown in Table 5 along with the power curves for the b path from which the coefficients corresponding to power levels can be read in Fig. 3. By Fritz and Mckinnon’s schema, we would expect the moderated a path effect size to be large and the b path to be medium, ranging small-medium to medium-large.

**Table 5:** Power scenarios for moderated mediation

*N* Clusters	*N* individuals	Design effect	Missing	Power a	Power b	Overall power	a	b
46	552	1.79	8%	0.81	0.99	0.80	0.65	0.49
46	552	1.79	8%	0.85	0.94	0.80	0.68	0.40
46	552	1.79	8%	0.9	0.89	0.80	0.74	0.36
46	504	1.73	15%	0.81	0.99	0.80	0.66	0.50
46	504	1.73	15%	0.85	0.94	0.80	0.70	0.41
46	504	1.73	15%	0.9	0.89	0.80	0.75	0.34
46	552	1.34	8%	0.81	0.99	0.80	0.56	0.43
46	552	1.34	8%	0.85	0.94	0.80	0.59	0.35
46	552	1.34	8%	0.9	0.89	0.80	0.65	0.31
46	504	1.31	15%	0.81	0.99	0.80	0.58	0.44
46	504	1.31	15%	0.85	0.94	0.80	0.62	0.36
46	504	1.31	15%	0.9	0.89	0.80	0.67	0.33

### Data management and analysis

#### Data management

Pseudonymised survey data will be collected using tablets via an online platform and saved on secure servers. Information linking participant IDs to their personal information and any data that contains participant information (e.g., audio recordings, transcripts) will be kept on a secure server or a password-protected computer. Hard copies that contain participant information (e.g., consent forms) will kept in locked cabinets. Audio recordings of interview and focus group discussions will be transcribed in full by the research team and securely transferred to translators. The translations will be anonymised, stored on password protected devices and transferred securely to researchers involved in the analysis. All reporting will be anonymised. To facilitate any secondary analyses, we will retain anonymised data for 20 years after the study as per MRC guidance.

#### Qualitative analysis

To analyse qualitative data pertaining to mechanisms of change and contextual factors, we will use dimensional analysis. This is a variant of grounded theory useful for refining CMOCs because of its focus on identifying and organising dimensions of experience — meanings, processes, conditions and consequences [67]. For analyses of implementation data, we will use the thematic analysis. We will develop a coding framework with deductive codes informed by topic guides and also allow codes to emerge from the data (inductive coding). Where possible, the researcher who conducted the interviews/focus group discussions will be involved in the transcription and analysis, and we will conduct member checks to validate the findings.

#### Quantitative analysis

A detailed, pre-specified statistical analysis plan will be finalised and approved by the Data Monitoring Committee (DMC) and the independent statistician of the Trial Steering Committee (TSC) before the database lock. The plan will be uploaded to the ISRCTN website. Reporting of results will adhere to the relevant Consolidated Standards of Reporting Trials (CONSORT) guidelines. We define the target treatment effect as follows:

- Population: 13- to 19-year-old adolescents from a randomised cluster (public mixed gender secondary school) who meet the trial inclusion/exclusion criteria and are identified as depressed by the PHQ-A (scoring 11 or more).
- Outcome: Depression scores at endline, 17 weeks post randomisation.
- Intervention: IPT compared to enhanced treatment as usual irrespective of treatment being discontinued (Intention to Treat, ITT).
- Population level summary measure: Individual mean PHQ-A score at 17 weeks post randomisation for each trial arm.
- Comparison: Mean difference between the trial arms at the participant-level at endline, 17 weeks post randomisation.

Following the principle of intention to treat (ITT) means adherence or compliance to allocated treatment is ignored and participants are followed up and analysed as part of their allocated groups. In this approach, data are collected after treatment discontinuation and missing data are included as if it was observed. The pattern of withdrawals in each trial arm will be assessed and any necessary remedial action will be approved by the TSC.

A descriptive summary of demographic and clinical variables at baseline and primary and secondary outcomes at baseline and post-randomisation (midline, endline and follow-up timepoints) will be reported by intervention group and overall. Mean, SD, medians and IQR will be used to summarise continuous variables and frequencies and percentages categorical variables respectively. Variables will be summarised at the cluster and individual level as appropriate.

The primary outcome of interest, the mean PHQ-A difference between the arms and 95% confidence intervals, will be estimated using mixed effects linear regression to take account of both the clustering due to the unit of randomisation being school and repeated measures by individual. The midline, endline and follow-up measures of the outcome measure are the dependent variable with a random intercept for cluster/school and an unstructured covariance matrix at the level of participant residuals to allow for repeated measures. This mmrm approach is preferred as it avoids model misspecification and is unbiased when data is missing at either random or completely at random [68]. The model will include the baseline measure of PHQ-A (ANCOVA approach), a dummy variable for trial arm, for timepoint and a trial arm by timepoint interaction to allow the treatment effect to vary across time. As randomisation is stratified by wave, this will always be included as a covariate in the analysis model. The model will be estimated using restricted maximum likelihood (REML) allowing the inclusion of all participants, with missing data as ignorable under the assumption of missing at random. Continuous secondary outcomes will be analysed using the same methods. Standardised effect sizes will be calculated by dividing the treatment arm difference estimate by the baseline standard deviation.

Model assumptions will be verified; linear mixed effects models assume residuals are normally distributed at the individual and cluster level and these will be checked appropriate data transformations will be applied if necessary and/or bootstrap procedures appropriate for multilevel models.

Model assumptions will be verified; linear mixed effects models assume that residuals are normally distributed at the individual and cluster level, which will be checked prior to analysis. Appropriate data transformations and/or bootstrap procedures appropriate for multilevel models will be applied if necessary.

To assess and report missing data, we will adhere to CONSORT guidelines. This includes presenting a table comparing baseline characteristics of patients with and without follow-up data, stratified by treatment arm, to identify potential bias. We do not expect any missing data at baseline, but if there is substantive missing data we will consider using multiple imputation by chained equations to address this. Missing data in the outcomes is accounted for by the designated mixed model estimated using restricted maximum likelihood estimation, which is valid under the ‘missing at random’ (MAR) assumption. This allows us to include all participants with at least one follow-up measurement to satisfy the ITT principle. To substantiate the MAR assumption, we will examine various baseline factors (e.g., demographics, clinical characteristics) as potential predictors of missingness and include significant predictors in the analysis model to improve its accuracy. As a sensitivity analysis, we will use multiple imputation with a substantive model which is consistent with the primary analysis model described above. Additionally, with multiple imputation we can consider using plausible ranges of patterns of missing data which allow departure from the MAR assumption based on the protocol of White et al., 2011 [69].

There are several planned additional secondary analyses of the primary outcome. To assess the effect of potential chance imbalance at baseline, effects of the size of grade 8- or 9-year groups per school will be controlled for and included as an additional covariate in the analysis models. Any changes in treatment effect will be noted. We will address the impact of treatment adherence using per protocol and Complier Average Causal Effects. In further secondary analysis we will evaluate CMOCs using planned multilevel mediation, moderation and moderated mediation analysis, noting that moderators (and potentially mediators) can occur at the cluster or individual level. These analyses are listed in Table 4.

#### Economic analysis

We will conduct a cost-utility analysis (CUA) and report an incremental cost-utility ratio (ICUR) reflecting the incremental cost per additional QALY gained by participating in the intervention, with and without discounting. Uncertainty will be characterised by probabilistic sensitivity analyses, varying cost inputs in 10,000 Monte Carlo simulations and assessing the probability of IPT being cost-effective at various willingness-to-pay thresholds. The time horizon for accumulating both costs and outcomes will be 30 months (the length of the full trial as well as follow-up) using 1-month time steps, capturing the longest possible timeframe for the intervention effects for which data collection is feasible. An annual discount rate of 3% will be applied to both costs and effects occurring beyond 12 months.

Subsequently we will conduct a benefit-cost analysis (BCA) to provide additional insights relevant for policy considerations. The BCA will use two approaches to valuing outcomes: (1) a human capital valuation approach using Nepal’s national minimum wage, and (2) a valuation of statistical life year approach (VSLY) for Nepal. Each approach will compare the net present value (NPV) of the QALYs gained in each treatment arm. Benefit-cost ratios and net benefits will be calculated for each approach. The probability of intervention vs. control conditions’ having positive NPV under each valuation approach will then be calculated via probabilistic sensitivity analysis with 10,000 replications.

#### Public and policy involvement

Our protocol is informed by several stakeholder groups. We adapted IPT for adolescents in Nepal through formative research with adolescents, parents, teachers and health workers in Nepal. We assessed the feasibility and acceptability of the intervention through qualitative interviews with the same stakeholder groups.

In the pilot cRCT, we piloted methods for this phase III cRCT and sought feedback from stakeholders on their acceptability. The interim qualitative and quantitative findings from the trial were presented to our TSC consisting of local Nepali and international experts. These findings informed the study procedures of the current protocol (e.g., screening procedures, inclusion and exclusion criteria, safety protocols, survey timing, survey battery, mechanism hypotheses). The same TSC will continue to meet throughout the Phase III trial. We will also convene a DMC to monitor the progress of the trial.

To input on the protocol and advise on trial issues, we have convened a youth advisory board in the study setting comprising ~ 8 local adolescent boys and girls who participated in the pilot cRCT.

#### Ethical issues

We have ethical approval from the Nepal Health Research Council (approval no. 2144) and University College London Research Ethics Committee (project ID 0351).

At the school level, we will seek permission to conduct the study from the school principal. Eligible adolescents who wish to participate in the trial will be asked to take home a written information sheet and consent form. We will obtain written consent from adolescents which will be collected before the baseline survey is conducted. For adolescents aged 17 and younger, we will take consent from their caregiver and adolescent assent. We will obtain consent for all adult participants from whom we collect data (e.g. teacher, IPT facilitator, parent).

We anticipate disclosures of suicidality at screening and throughout the trial. We have therefore developed a standard operating procedure (SOP) to manage these cases. This involves assessment by a psychosocial counsellor and referral to local mental health services where required. There is a separate SOP for disclosures of abuse which includes safety assessment and planning and potential referral to the Women, Children and Social Inclusion Section attached to the local municipality, or to a hospital where physical and mental health problems can be further assessed and managed.

#### Dissemination

We will proactively share findings with schools and advisory boards by developing simple research summaries, supported by workshops where requested. In Chitwan and Kathmandu we will hold workshops to disseminate findings to policy makers and relevant organisations. We will disseminate findings to academic audiences through publication in international peer-reviewed journals, and presentation at conferences. We will also hold a webinar for global policy makers and academics.

#### Trial status

Participant recruitment started for Wave 1 on May 15th 2025. The expected date for completion of recruitment is 30th November 2026 (Wave 8). IPT started for Wave 1 participants in June 2025 and for Wave 2 participants in July 2025.

## Discussion

We will conduct the first realist cluster-randomised controlled trial of a psychological intervention in a LMIC and help to address a key research gap about how these interventions work and their long-term effects. Moreover, we will contribute to scant evidence for the effectiveness of school based psychological interventions in LMICs. If our trial shows IPT is cost-effective, we envisage the intervention could be scaled up through the education system in Nepal. IPT would therefore be a vital component of a comprehensive model of school-based mental health care, aligning with the National Mental Health Strategy and Action Plan 2020 which includes integrating mental health topics in school curricula and providing mental health training for teachers and parents. Through our realist trial design, we will build evidence-based mid-range theories about intervention processes and how they can be modified and shaped by context. Researchers and policymakers from outside Nepal can then use this information to decide whether IPT might also be useful and effective in their context.

Our study has various strengths. We build on extensive formative research, piloting and stakeholder engagement, which confirmed the feasibility of our intervention and study design. We will use rigorous methods to mitigate bias including conducting randomisation after the baseline survey to reduce selection bias, masking research assistants to mitigate detection bias and publishing our statistical analysis plan to prevent reporting bias.

The study also has limitations. First, implementing the trial in the Nepali education system is challenging. Schools often close at short notice due to staff strikes, heat and public holidays. These closures will impact the trial timeline, for example delaying randomisation, survey administration or IPT sessions, although schools in control and intervention arms are likely to be equally affected. Second, although cluster randomisation minimises contamination and is more acceptable and feasible than individual randomisation, it decreases the likelihood of balancing individual participant characteristics across trial arms. Third, in trials of psychological interventions, it is inherently impossible to mask participants to allocation. Intervention participants may show improved mental health outcomes because they know they are receiving IPT (known as the Hawthorne Effect), which could potentially lead to overestimation of intervention effects. To mitigate this effect, we will match the survey burden across trial arms to the extent that it is possible, conduct long term follow-up to explore whether effects persist beyond the trial period and use objective outcome measures where feasible including school attendance and examination grades. Fourth, trials of psychological interventions with treatment as usual or waitlist controls have been criticised because these control conditions do not match hours of therapist contact in the intervention arm and it is therefore impossible to test whether an intervention is effective because of its specific techniques or because of generic effects such as supportive human contact [70]. To address this criticism, we will implement an enhanced control condition with assessment and referral for high-risk cases and capacity building in local clinics. Moreover, our realist design will enable us to examine mechanisms through which IPT improves depression including specific techniques and general elements.

In conclusion, this realist cRCT of school-based group IPT has the potential to generate robust evidence for the effectiveness of psychological interventions in Nepal and will help to inform translation and optimisation of these interventions in other low-resource settings.

## Data Availability

No datasets were generated or analysed during the current study.

## Abbreviations

95% CI: 95% confidence interval
CMOCs: Context–mechanism–outcome contingencies
(c)RCT: (Cluster) randomised trial
CSSRS: Columbia Suicide Severity Rating Scale
DMC: Data Monitoring Committee
DSRS: Depression Self–Rating Scale
EQ: 5D–EuroQol–5 Dimension 5 levels
GAD: 7–Generalised Anxiety Disorder–7
IPT: Interpersonal psychotherapy
IPT: A–Interpersonal psychotherapy modified for adolescents
ITT: Intention to treat
LMICs: Low–and middle–income countries
mhGAP: WHO mental health GAP Action Programme
MRC: Medical Research Council
PCL: 5–Post–Traumatic Stress Disorder Checklist for DSM–5
PHQ: A–Patient Health Questionnaire modified for adolescents
PRIME: PRogramme for Improving Mental health care
PTSD: Post–traumatic stress disorder
SEE: School Education Exam
SES: Standardised effect size
SOP: Standard operating procedure
TPO Nepal: Transcultural Psychosocial Organization Nepal
TSC: Trial steering committee
UCL: University College London
UKRI: UK Research and Innovation
UNICEF: United Nations Children’s Fund
US: United States
WHO: World Health Organization

## References

[1] Racine N, et al. Global prevalence of depressive and anxiety symptoms in children and adolescents during COVID-19: A Meta-analysis. JAMA Pediatr. 2021;175(11):1142–50. 34369987 10.1001/jamapediatrics.2021.2482 PMC8353576

[2] Santomauro DF, et al. Global prevalence and burden of depressive and anxiety disorders in 204 countries and territories in 2020 due to the COVID-19 pandemic. Lancet. 2021;398(10312):1700–12. 34634250 10.1016/S0140-6736(21)02143-7 PMC8500697

[3] The Lancet P. Global burden of disease 2021: mental health messages. Lancet Psychiatry. 2024;11(8):573. 39025623 10.1016/S2215-0366(24)00222-0

[4] Clayborne ZM, Varin M, Colman I. Systematic review and Meta-Analysis: adolescent depression and Long-Term psychosocial outcomes. J Am Acad Child Adolesc Psychiatry. 2019;58(1):72–9. 30577941 10.1016/j.jaac.2018.07.896

[5] Goodman E, Whitaker RC. A prospective study of the role of depression in the development and persistence of adolescent obesity. Pediatrics. 2002;110(3):497–504. 12205250 10.1542/peds.110.3.497

[6] Johnson D, et al. Adult mental health outcomes of adolescent depression: A systematic review. Depress Anxiety. 2018;35(8):700–16. 29878410 10.1002/da.22777

[7] United Nations, Department of Economic and Social Affairs, Population Division. World population prospects 2024: Summary of results. 2024. Available at: https://population.un.org/wpp/assets/Files/WPP2024_Summary-of-Results.pdf.

[8] Barbui C, et al. Efficacy of psychosocial interventions for mental health outcomes in low-income and middle-income countries: an umbrella review. Lancet Psychiatry. 2020;7(2):162–72. 31948935 10.1016/S2215-0366(19)30511-5

[9] Zhou X, et al. Comparative efficacy and acceptability of psychotherapies for depression in children and adolescents: A systematic review and network meta-analysis. World Psychiatry. 2015;14(2):207–22. 26043339 10.1002/wps.20217 PMC4471978

[10] Singla DR, et al. Psychological treatments for the world: lessons from low- and middle-income countries. Ann Rev Clin Psychol. 2017;13:149–81. 28482687 10.1146/annurev-clinpsy-032816-045217 PMC5506549

[11] Wampold BE. How important are the common factors in psychotherapy? An update. World Psychiatry. 2015;14(3):270–7. 26407772 10.1002/wps.20238 PMC4592639

[12] Cuijpers P, Reijnders M, Huibers MJH. The role of common factors in psychotherapy outcomes. Ann Rev Clin Psychol. 2019;15(1):207–31. 30550721 10.1146/annurev-clinpsy-050718-095424

[13] Duffy F, Sharpe H, Schwannauer M. The effectiveness of interpersonal psychotherapy for adolescents with depression - a systematic review and meta-analysis. Child Adolesc Mental Health. 2019;24(4):307–17. 10.1111/camh.12342 32677350

[14] Petersen I, et al. A group-based counselling intervention for depression comorbid with HIV/AIDS using a task shifting approach in South africa: A randomized controlled pilot study. J Affect Disord. 2014;158:78–84. 24655769 10.1016/j.jad.2014.02.013

[15] Bolton P, et al. Interventions for depression symptoms among adolescent survivors of war and displacement in Northern uganda: a randomized controlled trial. JAMA. 2007;298(5):519–27. 17666672 10.1001/jama.298.5.519

[16] World Health Organization and Columbia University. Group interpersonal therapy (IPT) for depression. (WHO generic field-trial version1.0). Geneva: WHO; 2016. https://www.who.int/publications/i/item/group-interpersonal-therapy-for-depression.

[17] Hassan E et al. Community perspectives on the implementation of a group psychological intervention for adolescents with de pression: A qualitative study in rural Nepal. Front Psychiatry. 2022;13. https://www.frontiersin.org/journals/psychiatry/articles/10.3389/fpsyt.2022.949251/full 10.3389/fpsyt.2022.949251 PMC9634215 36339866

[18] Rose-Clarke K et al. Culturally and developmentally adapting group interpersonal therapy for adolescents with depression in rural Nepal. BMC Psychol. 2020;8. https://link.springer.com/article/10.1186/s40359-020-00452-y 10.1186/s40359-020-00452-y PMC7425581 32787932

[19] Rose-Clarke K, et al. School-based group interpersonal therapy for adolescents with depression in rural nepal: a mixed methods study exploring feasibility, acceptability, and cost. Global Mental Health. 2022;9:416–28. 36618751 10.1017/gmh.2022.46 PMC9806967

[20] Bonell C, et al. Realist randomized controlled trials: A new approach to evaluating complex public health interventions. Soc Sci Med. 2012;75:2299–306. 22989491 10.1016/j.socscimed.2012.08.032

[21] Pawson R, Tilley N. Realistic evaluation. London: SAGE; 1997.

[22] Jamal F, et al. The three stages of Building and testing mid-level theories in a realist RCT: a theoretical and methodological case-example. Trials. 2015;16(1):466. 26470794 10.1186/s13063-015-0980-y PMC4608279

[23] Nielsen SB, Jaspers SØ, Lemire S. The curious case of the realist trial: methodological oxymoron or unicorn? Evaluation. 2024;30(1):120–37.

[24] Warren EA, Melendez-Torres GJ, Bonell C. Are realist randomised controlled trials possible? A reflection on the INCLUSIVE evaluation of a whole-school, bullying-prevention intervention. Trials. 2022;23(1):82. 35090541 10.1186/s13063-021-05976-1 PMC8796527

[25] Atmore KH, Bonell C, Luitel NP, Pradhan I, Shrestha P, Verdeli H, Rose-Clarke K. Exploring context, mechanisms and outcomes in group interpersonal therapy for adolescents with depression in Nepal: a qualitative realist analysis. Glob Ment Health (Camb). 2025 Feb 18;12:e19. 10.1017/gmh.2024.127. 10.1017/gmh.2024.127 PMC11867818 40028390

[26] Lipsitz JD, Markowitz JC. Mechanisms of change in interpersonal therapy (IPT). Clin Psychol Rev. 2013;33(8):1134–47. 24100081 10.1016/j.cpr.2013.09.002 PMC4109031

[27] Ravitz P, Maunder R, McBride C. Attachment, contemporary interpersonal theory and IPT: an integration of theoretical, clinical, and empirical perspectives. J Contemp Psychother. 2008;38(1):11–21.

[28] Tajfel, H., & Turner, J. C. An integrative theory of intergroup conflict. In: Austin WG, Worchel S, editors. The social psychology of intergroup relations. Monterey, CA: Brooks/Cole; 1979. p. 33–37. .

[29] Sapkota RP, Brunet A, Kirmayer LJ. Characteristics of adolescents affected by mass psychogenic illness outbreaks in schools in nepal: A Case-Control study. Front Psychiatry. 2020;11:493094. 33312130 10.3389/fpsyt.2020.493094 PMC7704439

[30] Posner K, et al. The Columbia-Suicide severity rating scale: initial validity and internal consistency findings from three multisite studies with adolescents and adults. Am J Psychiatry. 2011;168(12):1266–77. 22193671 10.1176/appi.ajp.2011.10111704 PMC3893686

[31] Nepal Statistics Office. National population and housing census 2021. Thapathali, Kathmandu: Government of Nepal; 2021.

[32] Bhatta P, Pherali T. Nepal: patterns of privatisation in education. A case study of low-fee private schools and private chain schools. Education International Research; 2017. https://www.right-to-education.org/sites/right-to-education.org/files/resource-attachments/2017_Education_International_Research_Nepal_ENG.pdf

[33] Pradhan U, Valentin K. Free education? Blurred public-private boundaries in state-run schooling in Nepal. Stud Nepali History Soc. 2020;25(2):277–98.

[34] Gautam P, et al. Depression among adolescents of rural nepal: A Community-Based study. Depress Res Treat. 2021;2021(1):7495141. 33628501 10.1155/2021/7495141 PMC7880710

[35] Solmi M, et al. Age at onset of mental disorders worldwide: large-scale meta-analysis of 192 epidemiological studies. Mol Psychiatry. 2022;27(1):281–95. 34079068 10.1038/s41380-021-01161-7 PMC8960395

[36] Kohrt BA, et al. Therapist competence in global mental health: development of the enhancing assessment of common therapeutic factors (ENACT) rating scale. Behav Res Ther. 2015;69:11–21. 25847276 10.1016/j.brat.2015.03.009 PMC4686771

[37] Pedersen G, et al. Developing the group facilitation assessment of competencies tool for group-Based mental health and psychosocial support interventions in humanitarian and Low-Resource settings. J Educ Emergencies. 2021;7(2):335–76.

[38] Johnson JG, et al. The patient health questionnaire for adolescents: validation of an instrument for the assessment of mental disorders among adolescent primary care patients. J Adolesc Health. 2002;30(3):196–204. 11869927 10.1016/s1054-139x(01)00333-0

[39] Luitel NP, et al. Translation, cultural adaptation and validation of patient health questionnaire and generalized anxiety disorder among adolescents in Nepal. Child Adolesc Psychiatry Ment Health. 2024;18(1):74. 38898474 10.1186/s13034-024-00763-7 PMC11188246

[40] Spitzer RL, et al. A brief measure for assessing generalized anxiety disorder: the GAD-7. Arch Intern Med. 2006;166(10):1092–7. 16717171 10.1001/archinte.166.10.1092

[41] Bolton P, Tang AM. An alternative approach to cross-cultural function assessment. Soc Psychiatry Psychiatr Epidemiol. 2002;37(11):537–43. 12395144 10.1007/s00127-002-0580-5

[42] Price M, et al. Investigation of abbreviated 4 and 8 item versions of the PTSD checklist 5. Psychiatry Res. 2016;239:124–30. 27137973 10.1016/j.psychres.2016.03.014

[43] Kane JC, Luitel NP, Jordans MJD, Kohrt BA, Weissbecker I, Tol WA. Mental health and psychosocial problems in the aftermath of the Nepal earthquakes: findings from a representative cluster sample survey. Epidemiol Psychiatr Sci. 2018 Jun;27(3):301–310. 10.1017/S2045796016001104. 10.1017/S2045796016001104 PMC5502203 28065208

[44] Kohrt BA, et al. Cross-cultural gene- environment interactions in depression, post-traumatic stress disorder, and the cortisol awakening response: FKBP5 polymorphisms and childhood trauma in South Asia. Int Rev Psychiatry. 2015;27(3):180–96. 26100613 10.3109/09540261.2015.1020052 PMC4623577

[45] Thapa SB, Hauff E. Psychological distress among displaced persons during an armed conflict in Nepal. Soc Psychiatry Psychiatr Epidemiol. 2005;40(8):672–9. 16021344 10.1007/s00127-005-0943-9

[46] The EuroQol Group. EuroQol-a new facility for the measurement of health-related quality of life. Health Policy. 1990;16(3):199–208. 10109801 10.1016/0168-8510(90)90421-9

[47] Little AW, Azubuike OB. Young lives school surveys 2016–17: the development of Non-Cognitive instruments in ethiopia, India and Vietnam. Editor: Young Lives; 2017.

[48] Marsh HW, et al. OECD’s brief self- report measure of educational psychology’s most useful affective constructs: Cross-cultural, psychometric comparisons across 25 countries. Int J Test. 2006;6(4):311–60.

[49] Herth P. Measuring hope: development of the herth hope index. J Nurs Scholarsh. 2000;32:309–15.

[50] Gratz KL, Roemer L. Multidimensional assessment of emotion regulation and dysregulation: development, factor structure, and initial validation of the difficulties in emotion regulation scale. J Psychopathol Behav Assess. 2004;26:41–54.

[51] Schwarzer R, Jerusalem M. Generalized Self-Efficacy scale. Measures in health psychology: A user’s portfolio. Causal and control beliefs. NFER-NELSON: Windsor, England; 1995. pp. 35–7. J. Weinman, S. Wright, and M. Johnston, Editors.

[52] Zimet GD, et al. The multidimensional scale of perceived social support. J Pers Assess. 1988;52(1):30–41. 10.1080/00223891.1990.9674095 2280326

[53] Buhrmester D, Furman W. The network of relationships inventory: Relationship qualities version. Unpublished measure, University of Texas at Dallas, 2008.

[54] Jordans MJD, et al. Effectiveness of group problem management plus, a brief psychological intervention for adults affected by humanitarian disasters in nepal: A cluster randomized controlled trial. PLoS Med. 2021;18(6):e1003621. 34138875 10.1371/journal.pmed.1003621 PMC8211182

[55] Sawyer M, et al. School-based prevention of depression: a randomized controlled study of the Beyondblue schools research initiative. J Child Psychol Psychiatry. 2010;51(2):199–209. 19702662 10.1111/j.1469-7610.2009.02136.x

[56] Global Early Adolescent Study. GEAS measures. Available from: https://geastudy.org/resources/geas-measures. [cited 2025 15th July].

[57] Goldberg DP, et al. The validity of two versions of the GHQ in the WHO study of mental illness in general health care. Psychol Med. 1997;27(1):191–7. 9122299 10.1017/s0033291796004242

[58] Frick PJ. The Alabama parenting questionnaire. Unpublished rating scale, University of Alabama, 1991.

[59] Burkey MD, et al. Validation of a cross-cultural instrument for child behavior problems: the disruptive behavior international Scale - Nepal version. BMC Psychol. 2018;6(1):51–51. 30390713 10.1186/s40359-018-0262-z PMC6215604

[60] Moore GF, et al. Process evaluation of complex interventions: medical research Council guidance. BMJ. 2015;350:h1258. 25791983 10.1136/bmj.h1258 PMC4366184

[61] May C. Towards a general theory of implementation. Implement Sci. 2013;8(1):18. 23406398 10.1186/1748-5908-8-18 PMC3602092

[62] Bonell C, Melendez-Torres GJ, Warren E. Realist trials and systematic reviews: rigorous, useful evidence to inform health policy. Cambridge: Cambridge University Press; 2024.

[63] Bonell C, et al. Dark logic’: theorising the harmful consequences of public health interventions. J Epidemiol Commun Health. 2015;69(1):95. 10.1136/jech-2014-204671 25403381

[64] Bonell C, et al. Effects of the learning together intervention on bullying and aggression in english secondary schools (INCLUSIVE): a cluster randomised controlled trial. Lancet. 2018;392(10163):2452–64. 30473366 10.1016/S0140-6736(18)31782-3 PMC6286420

[65] Jyani G, et al. Development of an EQ-5D value set for India using an extended design (DEVINE) study: the Indian 5-Level version EQ-5D value set. Value Health. 2022;7:1218–26. 10.1016/j.jval.2021.11.1370 35779943

[66] Fritz MS, Mackinnon DP. Required sample size to detect the mediated effect. Psychol Sci. 2007;18(3):233–9. 17444920 10.1111/j.1467-9280.2007.01882.x PMC2843527

[67] Bonell C, Warren E, Melendez-Torres G. Methodological reflections on using qualitative research to explore the causal mechanisms of complex health interventions. Evaluation. 2022;28(2):166–81.

[68] Bell ML, Rabe BA. The mixed model for repeated measures for cluster randomized trials: a simulation study investigating bias and type I error with missing continuous data. Trials. 21:148 (2020). 10.1186/s13063-020-4114-9 . 10.1186/s13063-020-4114-9 PMC7006144 32033617

[69] White IR. Strategies for handling missing data in randomised trials. Trials. 2011;12:A59.

[70] Argyris S, et al. Comparing apples and oranges in youth depression treatments? A quantitative critique of the evidence base and guidelines. BMJ Mental Health. 2025;28(1):e301162. 10.1136/bmjment-2024-301162 PMC11752052 39832835

